# Preparation of palladated porous nitrogen-doped carbon using halloysite as porogen: disclosing its utility as a hydrogenation catalyst

**DOI:** 10.1038/s41598-020-59003-5

**Published:** 2020-02-06

**Authors:** Samahe Sadjadi, Masoumeh Malmir, Giuseppe Lazzara, Giuseppe Cavallaro, Majid M. Heravi

**Affiliations:** 1 Gas Conversion Department, Faculty of Petrochemicals, Iran Polymer and Petrochemicals Institute, PO Box 14975-112, Tehran, Iran; 2 Department of Chemistry, School of Science, Alzahra University, PO Box 1993891176, Vanak, Tehran Iran; 3 Dipartimento di Fisica e Chimica, Università degli Studi di Palermo, Viale delle Scienze, pad. 17, 90128 Palermo, Italy; 4 Consorzio Interuniversitario Nazionale per la Scienza e Tecnologia dei Materiali, INSTM, Via G. Giusti, 9, I-50121 Firenze, Italy

**Keywords:** Chemistry, Materials science

## Abstract

In this article, halloysite nanoclay (Hal) was used as porogen for the synthesis of nitrogen doped porous carbon material with high specific surface area and pore volume. To this purpose, polymerization of melamine and terephthalaldehyde (MT) was performed in the presence of amine-functionalized carbon coated Hal (Hal@Glu-2N) that was prepared from hydrothermal treatment of Hal and glucose. Then, the prepared nanocomposite was palladated and carbonized to afford Pd@Hal@C. To further improve the textural properties of the nanocomposite, and introduce more pores in its structure, Hal nanotubes were etched. The characterization of the resulting compound, Pd@C, and comparing it with Pd@Hal@C, showed that etching of Hal significantly increased the specific surface area and pore volume in Pd@C. Pd@C was successfully used as a heterogeneous catalyst for promoting hydrogenation of nitroarens in aqueous media using hydrogen with atmospheric pressure as a reducing agent. The comparison of the structural features and catalytic activity of the catalyst with some control catalysts, including, Pd@Hal, Pd@Hal@Glu, Pd@Hal@Glu-MT and Pd@Hal@C confirmed that nitrogen groups in C could improve the Pd anchoring and suppress its leaching, while etching of Hal and introduction of more pores could enhance the catalytic activity through facilitating the mass transfer.

## Introduction

Carbon materials are among the most used compounds for diverse range of applications such as electrode materials, energy storage, catalysis and water treatment^1–5^. The function of carbon materials is strongly dependent on their physical and chemical properties such as specific surface area, porosity and the presence of heteroatoms in the carbon structure. According to the literature, doping of heteroatoms such as nitrogen atoms in the carbon structure can improve the electric and chemical properties of carbon materials and expand their applications^2,3,6–10^. To achieve nitrogen doped carbon, carbonization of heteroatom-containing substrates has been suggested. However, the control of the textural properties of the carbon material through simple carbonization is challenging. To furnish a solution to this issue, use of template and structural guides such as KIT and mesoporous silica has been suggested^11,12^. However, most of the templates are synthetic and use of them is costly and lingers the synthetic process of carbon materials^13^.

Halloysite is a natural clay mineral (imperial formula of Al_2_Si_2_O_5_(OH)_4_·nH_2_O) with tubular morphology and chemistry similar to Kaolin^14–23^. This nanoclay has been successfully applied for various applications such as material chemistry, catalysis and smart delivery^18,24–26^. It should be noted that the specific geological origin of Hal affects the morphological features and size polydispersity of nanotubes^27^. Moreover, the chemistry of the surface of Hal is readily tuned-able and can be modified through surface functionalization^15,16,27^.

Reduction of nitro group to amine functionality is an industrially attractive chemical transformation that is mostly accomplished in the presence of hydrogen gas as reducing agent and precious metals as catalysts^28^. Using this reaction, diverse range of chemicals such as anilines can be synthesized. The precious metals are mostly immobilized on the convenient catalyst supports to afford heterogeneous catalysts^29–32^. As the support may affect the catalytic activity and/or suppress the leaching of the precious metal, wise choice of the catalyst support is imperative.

In the continuation of our interest in disclosing the utility of Hal^33–37^, we have reported synthesis of Hal-melamine based polymer hybrid system^38^. Moreover, we successfully developed several hybrid of Hal-carbon materials that exhibited excellent catalytic activity^37,39^. Taking these results into account and considering the fact that melamine-based polymers possess high content of nitrogen^40^, in this study, we aim to use Hal as porogen for tuning the textural features of nitrogen doped carbon materials. To the best of our knowledge, although Hal was previously used for the synthesis of carbon nanotubes^41^, there is no report on the use of Hal as a porogen for improving porosity of carbon sheets. In this study, melamine-based mesoporous polymer network as a carbon precursor was formed in the presence of amine-functionalized Hal coated with glucose-derived carbon. Subsequently, the resulting nanocomposite was applied for the immobilization of Pd nanoparticles. The latter was then carbonized to afford palladated carbon material that contained Hal. To modify the surface properties of the carbon material and introducing more porosity, Hal was etched by washing with HF. The resulting catalyst, Pd@C, exhibited excellent catalytic activity, selectivity and recyclability for the hydrogenation of nitroarene. Using several control catalysts and comparing their Pd loading, leaching, specific surface area and catalytic activity with those of the catalyst, the roles of carbonization and Hal etching in the catalytic activity were studied.

## Result and Discussion

### Catalyst characterization

The XRD pattern of each intermediate material that was obtained in the course of synthesis of Pd@C as well as the XRD pattern of the final catalyst were recorded and compared with that of pristine Hal, Figure S1. It was found that, the characteristic bands of Hal were appeared at 2*θ* = 11.5°, 20.2°, 26.7°, 35.4°, 55.6° and 62.1°, JCPDS No. 29–1487 (labelled as *))^42,43^. In the XRD pattern of Hal@Glu, the Hal characteristic bands were observed. However, their intensities decreased remarkably. This observation is in good accord with the previous reports^44^. In the XRD pattern of Pd@Hal@C, three types of characteristic bands were detected. First, the characteristic bands of Pd nanoparticles (the bands at 2θ = 41.1 °, 45.5°, 68.7°, 79.6°, and 87°, labelled as P) that can be assigned to the {111}, {200}, {220}, {311} and {222} planes of Pd (JCPDS, No.46-1043))^45^. Moreover, the characteristic bands of Hal with very low intensities were observed. According to the literature, upon thermal treatment, Hal begins to destruct and its characteristic bands start showing lower intensities and disappearing^44,46^. In the XRD pattern of Pd@Hal@C, this issue is observable. However, the presence of Glu shell that surrounded the Hal tubes render them more stable against thermal treatment. Therefore, the Hal characteristic bands can be still detected^44^. The third type of the characteristic bands in the Pd@Hal@C XRD pattern is the bands that can be assigned to the carbon material. More precisely, the bands at 2*θ* = 25.2°, 42.5°, 43.4°, 73.8°, can indicate the diamond–lonsdaleite system^47^. The disappearance of the small characteristic bands of Hal in the XRD pattern of Pd@C can confirm the successful etching of Hal. Furthermore, the observation of the characteristic bands of carbon can prove that the carbon material was stable in the course of etching.

FTIR spectroscopy was also applied to verify the formation of Pd@C and the compounds prepared in the course of synthesis of Pd@C, Figure S2. According to the literature^44^, the characteristic bands of pristine Hal can be listed as the bands at 3694 and 3627 cm^−1^ that can be assigned to the internal -OH functionality, 1031 cm^−1^ that is representative of Si–O stretching and 540 cm^−1^ that is due to the Al-O-Si vibration. The FTIR spectrum of glucose exhibited the characteristic bands at 3411 cm^−1^ (-OH functionality), 2942 cm^−1^ (-CH_2_) and 1461 cm^−1^. The FTIR spectrum of Hal@Glu exhibited the characteristic bands of Hal, implying that Hal preserved its structure upon hydrothermal treatment. The FTIR spectrum of Pd@C is significantly distinguished from that of Hal@Glu and does not show the characteristic bands of Hal. This observation is quite expectable, as Hal has been etched in the course of catalyst preparation. In the case of Pd@Hal@C, in which Hal is still present, some of the Hal characteristic bands are absent. The disappearance of some of the Hal characteristic bands is due to thermal treatment at high temperature.

**Figure 1:**
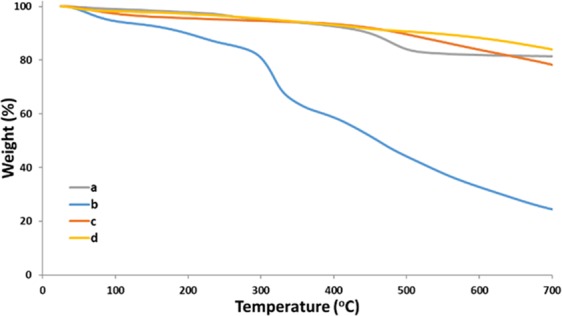
The TG analyses of (**a**) pristine Hal, (**b**) Hal@Glu-MT, (**c**) Pd@Hal@C, (**d**) Pd@C and Hal@Glu.

In Fig. 1, the TGA thermograms of pristine Hal, Hal@Glu-MT, Pd@Hal@C, Hal@Glu and Pd@C are compared. As shown, the pristine Hal possessed high thermal stability and showed only the weight loss due to the loss of structural water and Hal dehydroxylation (~160 °C and 550 °C respectively)^25,48^. On the contrary, Hal@Glu exhibited lower thermal stability. The comparison Hal@Glu with that of Hal indicates that apart from the weight losses of pristine Hal, an additional weight loss at 450 °C can be detected that can be attributed to the degradation of Glu. In the case of Hal@Glu-MT, one more weight loss step at 320 °C can be observed that indicates the successful formation of MT in the structure of the catalyst. Considering the thermograms of Pd@Hal@C and Pd@C, it can be concluded that these two samples exhibited significantly higher thermal stabilities compared to that of Hal@Glu-MT and Hal@Glu, confirming the successful carbonization.

Raman spectroscopy was also applied for the characterization of the prepared porous carbon material, Figure S3. The observation of two bands at 1359 (D-band) and 1605 cm^−1^ (G-band) can confirm the graphitic nature of the catalyst. In more detail, the D-band is indicative of the sp^3^ configuration due to the presence of intrinsic defects and the observed G-band can be attributed to the graphitic carbon^49–51^. Notably, the calculated I_D_/I_G_ was measured to be 0.83, confirming high amounts of defective or disordered graphitic structures^52^.

**Figure 2:**
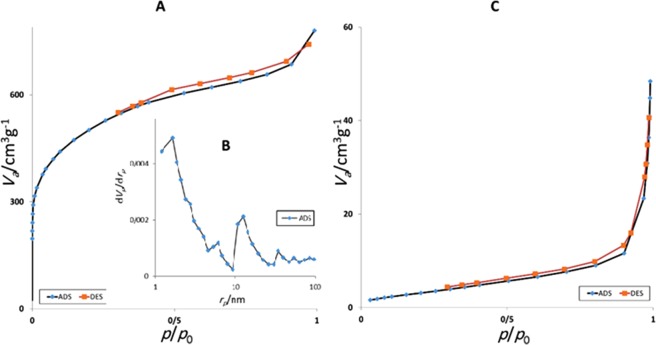
The N_2_ adsorption-desorption isotherms of Pd@C and Pd@Hal@C (**A** and **C**) and BJH plot of Pd@C (**B**).

In the next step, the effect of etching of Hal on the textural properties of the catalyst was studied. To this purpose, the N_2_ adsorption-desorption isotherms of Pd@Hal@C and Pd@C were recorded and compared, Fig. 2. As depicted, the isotherms of two samples are distinguished. More precisely, Pd@Hal@C exhibited type II isotherm, while Pd@C showed type IV. To further verify this issue, the specific surface area of two catalysts were calculated and compared, Table S1. It was found that, this value for Pd@C is significantly larger than that of Pd@Hal@C. The comparison of total pore volume of two samples also showed the effect of Hal etching. More precisely, this value for Pd@C was much higher than that of Pd@Hal@C, indicating the removal of Hal resulted in the formation of pores, Table S1. Moreover, the pore size distribution curves of the Pd@C was obtained by the BJH method. As shown, two types of pores, i.e. mesopores (2 nm) and micropores (~8 and 10.5 nm) were observed in Pd@C. Hence, it can be concluded that in the structure of the catalyst large pores exist that can facilitate the mass transfer.

**Figure 3:**
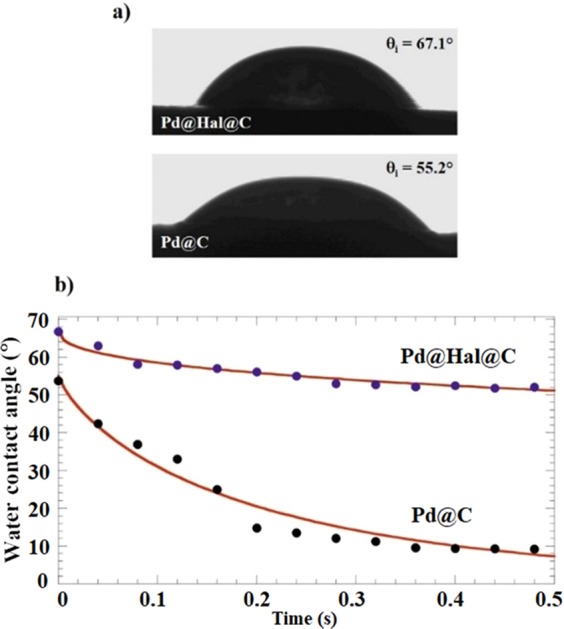
(**a**) Images of the water droplets just after their deposition on the surface of Pd@Hal@C and Pd@C. The corresponding θ_i_ are reported within the images. (**b**) The water contact angle as a function of time for Pd@Hal@C and Pd@C. The solid red line represents the fitting based on the equation 1.

Water contact angle experiments were also conducted on Pd@Hal@C and Pd@C in order to explore the influence of the Hal etching on the wettability properties of the nanomaterials. Figure 3a displays the images of the water droplets immediately after their deposition on the surface of the nanomaterials.

It was detected that Pd@Hal@C presents a hydrophobic surface being as evidenced by its initial contact angle (67.1°), which is much larger compared to that (20°) of pristine Hal^50^. These results indicated that the hydrophobic coverage of Hal outer surface induced by the synthetic procedure. As concerns Pd@C, a slight decrease of the initial water contact angle (55.2°) was observed. This value agrees with the carbonaceous composition of Pd@C. Further information on the wettability and structural characteristics of Pd@C and Pd@Hal@C were obtained by monitoring the time evolution of the contact angle (Fig. 3b). According to the literature^27,53^, the evolution of the water contact angle (θ) on time (t) can be described by the following empiric equation:

1 $${\rm{\theta }}={{\rm{\theta }}}_{i}\cdot \exp \,(\,-\,{k}_{\theta }\cdot {t}^{n})$$

Where θ_i_ corresponds to the initial contact angle, k_θ_ and n are fitting parameters related to the kinetics and the mechanism of the process. As shown in Fig. 3b, equation 1 was successful in the fitting of the θ vs t experimental trends. Accordingly, k_θ_ and n parameters were determined for both Pd@Hal@C and Pd@C (k_θ_ and n for Pd@C were 3.5 ± 0.4 s^−1^ and 0.78 ± 0.09 respectively, whereas these values for Pd@Hal@C were 0.364 ± 0.019 s^−1^ and 0.43 ± 0.05 respectively). In this line, n values were estimated between 0 and 1, highlighting that both absorption and spreading contribute to the θ decrease over time. Interestingly, k_θ_ is much larger (one order of magnitude) for Pd@C respect to that of Pd@Hal@C. The faster kinetic evolution of the contact angle might be attributed to the enhanced porosity of Pd@C evidenced by BET data.

Based on CHN elemental analysis, the percent of C, N, and H of the catalyst were calculated to be 68.31, 7.64, and 2.92 wt%, respectively. This result indicated that carbonization of Pd@Hal@Glu-MT result to N-doped carbon with relatively good nitrogen content.

**Figure 4:**
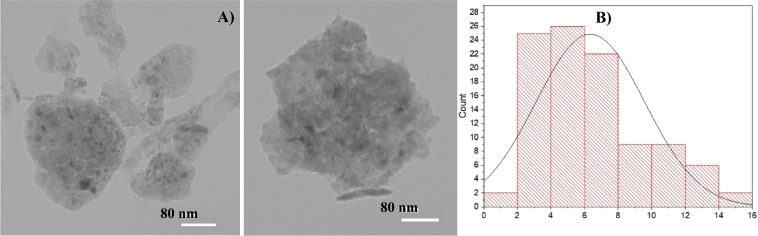
TEM images (**A**) and size distribution histogram (**B**) of the Pd@C.

In the following, the morphology of the catalyst was studied. In the TEM images of the catalyst, Fig. 4A, the carbon sheet can be observed. Furthermore, the tiny black spots that are indicative of Pd nanoparticles can be detected. It was also found out that the Pd nanoparticles were distributed on the porous carbon homogeneously. To estimate the average Pd particle size, the size of ~ 200 nanoparticles found in an arbitrarily chosen area of the TEM images were estimated via ImageJ program and the size distribution curve was drawn via Origin software, Fig. 4B. The Gaussian-fitted curve shows the mean diameter (d) as 6.34 nm with a standard deviation (r) of ±3.24 nm.

### Investigation of the catalytic activity

To initiate the investigation of the catalytic activity of Pd@C, hydrogenation of nitroarenes was targeted as a model Pd-catalyzed chemical transformation and among various substrates, nitrobenzene was considered as a model nitroarene. In the following, the reaction condition was optimized to achieve the highest yield of aniline as the desired product. To this purpose, the reaction variables (catalyst amount, reaction solvent and temperature) were varied and their effects on the yield of aniline were disclosed. The results showed that the optimum reaction condition was performing the hydrogenation reaction in water as environmentally-benign solvent, at 50 °C in the presence of 0.85 mol% Pd@C, under which quantitative conversion and yield could be achieved. In the next step, the scope of the reaction was examined to verify the generality of the presented method. In this line, substituted nitrobenzenes as well as 1-nitro naphthalene and 4-nitro acetophenone were applied as substrates, Table 1. As tabulated, Pd@C could successfully promote the hydrogenation reaction of sterically demanding 1-nitro naphthalene. However, the yield of the reaction was lower than that of small nitrobenzene. Moreover, 4-nitro acetophenone that possesses two unsaturated functionalities (-C=O and –NO_2_) just led to the reduction of nitro group in high yield, but not –C=O functionality, confirming the high selectivity of Pd@C towards hydrogenation of –NO_2_ functionality. In addition, it can be observed that the presence of electron-donating group (-NH_2_) led to decrease of the yield of the reaction due to the stabilization of resonance effects to the nitro functionality^54^. According to the literature, in the case of halogen substituted reagent, dehalogenation may be responsible for low yield^55^.

**Table 1:** Pd@C catalyzed Hydrogenation reaction of nitro arenes^a^.

Entry	Reagent	Product	Time (h:min)	Yield^b^ (%)
1			0:45	100
2			2:35	85
3			1:40	90
4			3:00	55
5			3:45	60

^a^Reaction condition: nitro arene (1 mmol), Pd@C (0.85 mol%) in water (5 mL) at 50 °C under 1 atm. H_2_ gas.

^b^Isolated yield.

In the following, the effects of carbonization of MT, Glu shell and Hal etching on the catalytic activity were studied. In this line, four control catalysts, including, Pd@Hal, Pd@Hal@Glu, Pd@Hal@Glu-MT and Pd@Hal@C were prepared (see the Experimental section) and their catalytic activities for promoting model reaction were examined and compared with that of Pd@C, Table 2. To provide more insight into the differences of the catalytic activity of the samples, their specific surface area and Pd loading and leaching were also measured and compared, Table 2.

**Table 2:** Comparison of the Pd loading and leaching and the catalytic activity of the present catalyst with the other prepared catalysts in the hydrogenation reaction^a^.

Entry	Catalyst	Yield^b^ (%)	Time (min)	Loading of Pd NPs (mmol/g)	Leaching of Pd NPs (mmol/g) after 10 recycling	*S*_BET_ (m^2^g^−1^)
1	Pd@C	100	45	0.052	0.0016	1761
2	Pd@Hal@C	95	45	0.044	0.0019	132
3	Pd@Hal@Glu-MT	70	45	0.042	0.0095	46
4	Pd@Hal@Glu	58	45	0.013	0.012	34
5	Pd@Hal	60	45	0.013	0.011	43

^a^Reaction condition: nitrobenzene (1 mmol), catalyst (0.85 mol%) in water (5 mL) at 50 °C under 1 atm. H_2_ gas.

^b^Isolated yield.

As shown in Table 2, the five samples showed not only different catalytic activities, but also different Pd loading, leaching and specific surface area. As tabulated, the catalytic activity decreased in the order of Pd@Hal@Glu < Pd@Hal < Pd@Hal@Glu-MT < Pd@Hal@C < Pd@C. The order of Pd loading is very similar to that of the catalytic activity (Pd@Hal@Glu = Pd@Hal < Pd@Hal@Glu-MT < Pd@Hal@C < Pd@C). Regarding the specific surface area, it can be seen that the specific surface areas of three samples of Pd@Hal@Glu, Pd@Hal and Pd@Hal@Glu-MT are in the same range (34–46 m^2^g^−1^), while Pd@Hal@C showed higher specific surface area (132 m^2^g^−1^). In the case of Pd@C this value significantly increased and reached to 1761 m^2^g^−1^.

This observation can indicate that the different structure of the catalyst can result in the different Pd loading and specific surface area and consequently catalytic activity. In more detail, in Pd@Hal@Glu that Hal surface is covered with Glu, the specific surface area is slightly reduced compared to Pd@Hal. However, the introduction of Glu did not affect the loading of Pd and two catalysts that possessed almost similar specific surface area and Pd loadings exhibited similar catalytic activity. Moreover, the Pd leaching of two samples are almost similar. As shown, the specific surface area of Pd@Hal@Glu-MT is comparable to that of Pd@Hal. However, the Pd loading in this sample is significantly higher than that of the aforementioned catalysts, implying that the presence of MT polymer that is enriched in nitrogen functionality can remarkably improve Pd anchoring. This can be achieved through the electrostatic interactions of nitrogen containing functionalities in MT with Pd nanoparticles. The higher Pd loading can justify the superior catalytic activity of this sample compared to the two previous ones. Moreover, comparing the Pd leaching of this sample with the two other samples confirms that MT can suppress Pd leaching. In the case of Pd@Hal@C, the specific surface area increased but the Pd loading is comparable to that of Pd@Hal@Glu-MT. This observation can indicate that carbonization and consequently increase of the specific surface area can lead to the enhancement of the catalytic activity. Notably, the Pd leaching in Pd@Hal@C was dramatically suppressed. Finally, in the catalyst, Pd@C, that possesses the highest specific surface area and Pd loading, the best catalytic activity was observed. In fact, apart from higher Pd loading, etching of Hal and introduction of more pores (see the BET results), can facilitate the mass transport in this sample. Moreover, this sample exhibited the best recyclability (vide infra).

### Hot filtration test

Next, the heterogeneous nature of the catalysis was verified by hot filtration method^56^. In this line, the model reaction was held after a short period of time and the catalyst was filtered off. Then, the hydrogenation was continued in the filtrate. To elucidate whether the reaction can proceed in the absence of the catalyst, the reaction was monitored. The lack of progress of the reaction confirmed the heterogeneous nature of the catalysis.

### Catalyst recyclability

To elucidate whether Pd@C can be considered as a recyclable catalyst, the recycling test was carried out for the model hydrogenation reaction. As described in the Experimental section, the recovered catalyst was washed and dried and re-used for the next run of hydrogenation reaction under exactly similar condition. The results of the recycling experiment up to ten reaction runs are illustrated in Figure S4. It was found that, Pd@C maintained its high catalytic activity on the second reaction run with no loss of the catalytic activity. Upon further recycling up to seven reaction runs, slight decrease in the catalytic activity was observed. Further recycling, however, led to recognizable loss of the catalytic activity and upon ten recycling the yield of aniline reached 69%.

**Figure 5:**
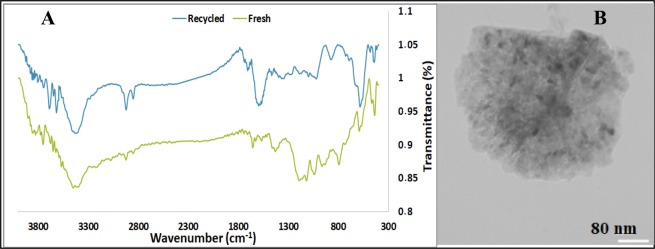
The FTIR spectra (**A**) of the fresh and recycled Pd@C and TEM image (**B**) of the recycled Pd@C after ten runs of the model reaction under optimum reaction condition.

To investigate the effects of recycling on the structure and morphology of the catalyst, FTIR spectra and TEM image of the recycled catalyst were recorded and compared with that of the fresh catalyst. The study of the catalyst recycled for ten reaction cycles via FTIR spectroscopy, Fig. 5A, showed that the FTIR spectrum of the recycled Pd@C exhibited the characteristic bands of the fresh Pd@C. However, there are some difference between two spectra. This may be due to the disposition of nitrobenzene on the surface of the catalyst. Notably, as tabulated in Table 2, the leaching of Pd nanoparticles after 10 reaction runs was negligible. Moreover, the TEM image of the reused catalyst was obtained and compared with that of the fresh catalyst, Fig. 5B. The similarity of both images could be considered as another proof for the stability of the reused catalyst. All these analyses can confirm that the catalyst was stable upon reusing and can be reused for the successive reaction runs. Considering these results, the drop of the catalytic activity after ten reaction runs was attributed to the coverage of C that is catalytically active with the reagent.

## Methods

### Synthesis of the catalyst

#### Synthesis of Hal@Glu

Inspired by the previous reports, Hal@Glu nanocomposite was synthesized by hydrothermal method^57^. First, a well dispersed suspension of Hal (1 g) in deionized water (40 mL) was prepared by using ultrasonic irradiation (power 100 W, 15 min). Next, glucose (6 g) was introduced in the obtained suspension and the mixture was transferred into a Teflon-lined stainless steel autoclave (150 mL). The container was then, sealed and maintained at 200 °C for one day. At the end of the hydrothermal treatment, the reactor was cooled down to room temperature and the product was filtered, rinsed with ethanol, centrifuged for five times and dried in oven at 80 °C.

#### Preparation of Hal@Glu-2N

The amine functionalized Hal@Glu was obtained by adding 3-*N*-(2 (trimethoxysilyl)ethyl)methanediamine (3 mL) as a functionalization group and Et_3_N (3 mL) as a catalyst in the stirring suspension of Hal@Glu (2.5 g) in toluene (50 mL), followed by refluxing at 140 °C overnight. Upon completion of the reaction, the resulting solid was filtered off, washed with toluene repeatedly and dried at 100 °C overnight.

#### Synthesis of Hal@Glu-MT

Hal@Glu contained melamine-based mesoporous polymer network was prepared based on the previous reported work with slight modification^58,59^. Briefly, 500 mL single-necked flask, fitted with a condenser and a magnetic stirring bar was charged with Hal@Glu-2N (1 g), melamine (2.485 mmol), terephthalaldehyde (3.728 mmol) and DMSO (15.5 mL). The flask was then completely deoxygenated by bubbling purified argon for 30 min. Upon heating the flask from ambient temperature to the boiling temperature in an oil bath, the polymerization process initiated and continued for 96 h. Upon completion of the reaction, the obtained Hal@Glu contained melamine-based mesoporous polymer network (Hal@Glu-MT) was filtrated off and washed with a mixture of acetone, THF and dichloromethane. Finally, the solvent was removed to afford the product as a gray powder.

#### Immobilization of Pd nanoparticles on Hal@Glu-MT

To immobilize Pd nanoparticles on the Hal@Glu-MT, a solution of Pd(OAc)_2_ (0.02 g) in MeOH (10 mL) was added to the solution of suspended Hal@Glu-MT (1 g) in toluene (50 mL) in a drop wise manner under stirring condition overnight. In the following, a solution of NaBH_**4**_ in MeOH (12 mL, 0.2 N) was added under inert atmosphere to the above-mentioned suspension. Subsequently, the resulting mixture was stirred for 6 h. The obtained Pd@Hal@Glu-MT, was filtrated off, washed with toluene and MeOH and dried in an oven at 80 °C for 12 h.

#### Synthesis of Pd@Hal@C

In this step, Pd@Hal@Glu-MT (5 g) was placed in a quartz container and heated up to 450 °C for 1 h under argon flow and heating rate of 30 °C min^−1^. The sample was held at this temperature for 6 h. After cooling to room temperature, the product (3 g) was recovered as a black powder.

#### Synthesis of Pd@C

**Figure 6:**
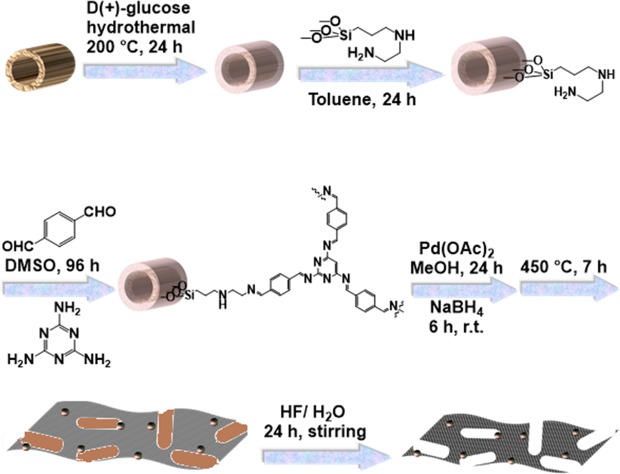
The procedure for the synthesis rout of the Pd@C.

For the removal of the Hal, etching method by using HF was applied. In this regard, the as synthesized Pd@Hal@C (3 g) was placed in an acid-resistant plastic vessel and then, 250 mL of HF solution was drop-wisely added and the mixture was allowed to cool to room temperature and kept in the acidic solution for 24 h. Upon completion of the etching process, the product was washed with distilled water to remove the acid. Upon reaching the neutral pH, the solid was collected and then dried in an oven at 80 °C overnight. The schematic procedure for the synthesis of the catalyst is illustrated in Fig. 6. Noteworthy, synthesis of control catalysts, Pd@Hal@Glu-MT, Pd@Hal@Glu, and Pd@Hal were performed in similar ways, except Hal@Glu-MT, Hal@Glu and Hal were used as catalyst support for Pd immobilization respectively. In the case of Pd@Hal@C, Hal was not etched and the catalyst contained Hal.

### Hydrogenation of nitrobenzene

In a typical procedure, Pd@C catalyst (0.85 mol%), nitrobenzene (1.0 mmol) and deionized water as an environmentally-benign solvent were placed in a flask (5 mL). The reaction vessel was then heated up to 50 °C and H_2_ (1 atm.) was purged as a reducing agent. Upon completion of the reaction (traced by TLC), the reactor was slowly cooled and depressurized and Pd@C was filtered off by simple filtration, washed with EtOH/H_2_O several times and dried in oven at 70 °C for 7 h. To afford the hydrogenated product, the solvent was evaporated and the resulting aniline was characterized by comparing its melting/boiling point as well as FTIR spectrum with those of the known compounds.

## Conclusion

A novel N-doped mesoporous carbon, Pd@C, was prepared through preparation of Pd@Hal@Glu-MT via coating of Glu carbon shell on Hal, functionalization, growth of MT polymer, Pd immobilization and carbonization, followed by etching of Hal. The resulting hybrid exhibited high catalytic activity and selectivity for the hydrogenation of nitroarenes. The comparison of the catalytic activity of Pd@C with that of Pd@Hal, Pd@Hal@Glu, Pd@Hal@C and Pd@Hal@Glu-MT, confirmed higher catalytic activity of the former. This result was attributed to the increasing the interactions of Pd with the nitrogen atoms of the support and consequently increasing of Pd loading as well as suppressing the Pd leaching. Moreover, as etching of Hal can dramatically increase the specific surface area and pore volume, the superior catalytic activity of Pd@C can also be assigned to the improved mass transport in Pd@C. The recyclability test confirmed high recyclability of Pd@C with slight Pd leaching and no Pd aggregation. Moreover, the hot filtration test indicated the heterogeneous nature of the catalysis.

## Supplementary information

Supplementary information.
