# Supplementary material for: Preparation of palladated porous nitrogen-doped carbon using halloysite as porogen: disclosing its utility as a hydrogenation catalyst
Source: Sci Rep. 2020 Feb 6;10:2039. doi: 10.1038/s41598-020-59003-5 (PMC7005045; doi:10.1038/s41598-020-59003-5)
Supplement: Supplementary file 1 — Supplementary information. [file 41598_2020_59003_MOESM1_ESM.docx]

**Supporting information**

- 1. **Materials**

All the chemicals and solvents used for the synthesis of Pd@C and the control catalysts, including Hal, 3-*N*-(2 (trimethoxysilyl)ethyl)methanediamine, melamine, terephthalaldehyde, NaBH_4_, Pd(OAc)_2_, D-(+)-glucose, Et_3_N, toluene, DMSO, MeOH, EtOH, hydrofluoric acid, deionized water and toluene were provided from Sigma-Aldrich. The hydrogenation reaction was performed by using nitroarenes. All was purchased from Sigma-Aldrich.

- 1. **Instrument**s

The characterization techniques used for the verification of the catalysts and all the control samples included, TGA, XRD, CHN, FTIR, BET, TEM, Raman, ICP-AES. Fourier transform infrared (FTIR) spectra were recorded on PERKIN-ELMER- Spectrum 65 instrument. Transmission electron microscope (TEM) images of the final catalyst were recorded using CM30300Kv field emission transmission electron microscope. X-ray diffraction (XRD) patterns were obtained by a Siemens D5000 85 diffractometer with Cu Kα radiation in 2θ range of 5-90°. Thermogravimetric analysis (TGA) under inert condition was performed at heating rate of 10 °C min^-1^ using METTLER TOLEDO instrument. BELSORP Mini II apparatus was applied for recording N_2_ adsorption-desorption isotherm of the catalyst (the sample preparation was carried out by preheating of the samples at 100 °C for 3 h). The used Raman spectrometer for analyzing the catalyst was TEKSAN-N1-541 Spectrum at k= 532 nm instrument. ICP analyzer used for measuring the Pd loading and leaching of the catalysts was Varian, Vista-pro. Water contact angle tests were conducted on Pd@Hal@C and Pd@C pellets by using an optical contact angle apparatus (OCA 20, Data Physics Instruments) equipped with a video measuring system having a high-resolution CCD camera and a high-performance digitizing adapter. Data acquisition was conducted by SCA 20 software (Data Physics Instruments). The contact angle (θ) of water in air was detected through the sessile drop method by placing a water droplet of 10 ± 0.5 mL onto the surface of nanomaterials tablets. The measurements were conducted at 30.0 ± 0.1 °C. The time evolution of the contact angle was investigated.

The facilities applied for the synthesis of the catalyst included, ultrasonic apparatus (Bandelin HD 3200 with output power between 100-200 W and tip TT13), Teflon-lined stainless steel autoclave (150 mL) and furnace equipped with inert gas.


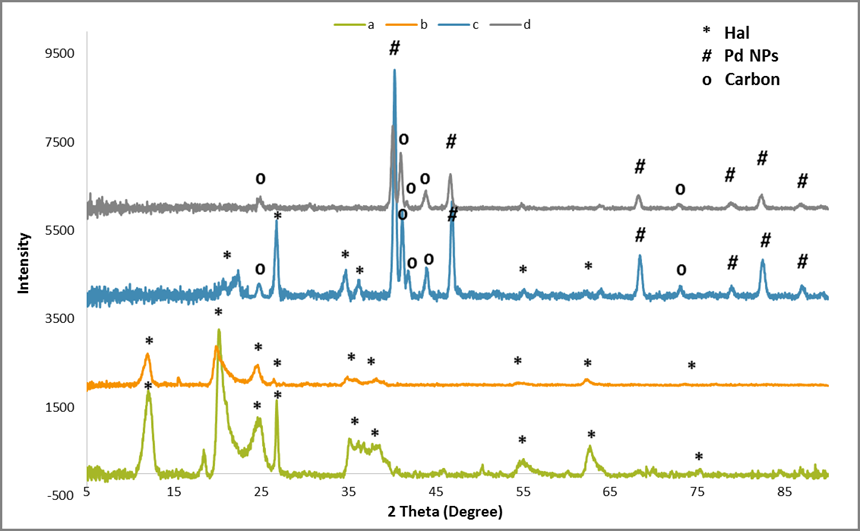


**Figure S1.** The XRD patterns of (a) pristine Hal, (b) Hal@Glu, (c) Pd@Hal@C and (d) Pd@C.


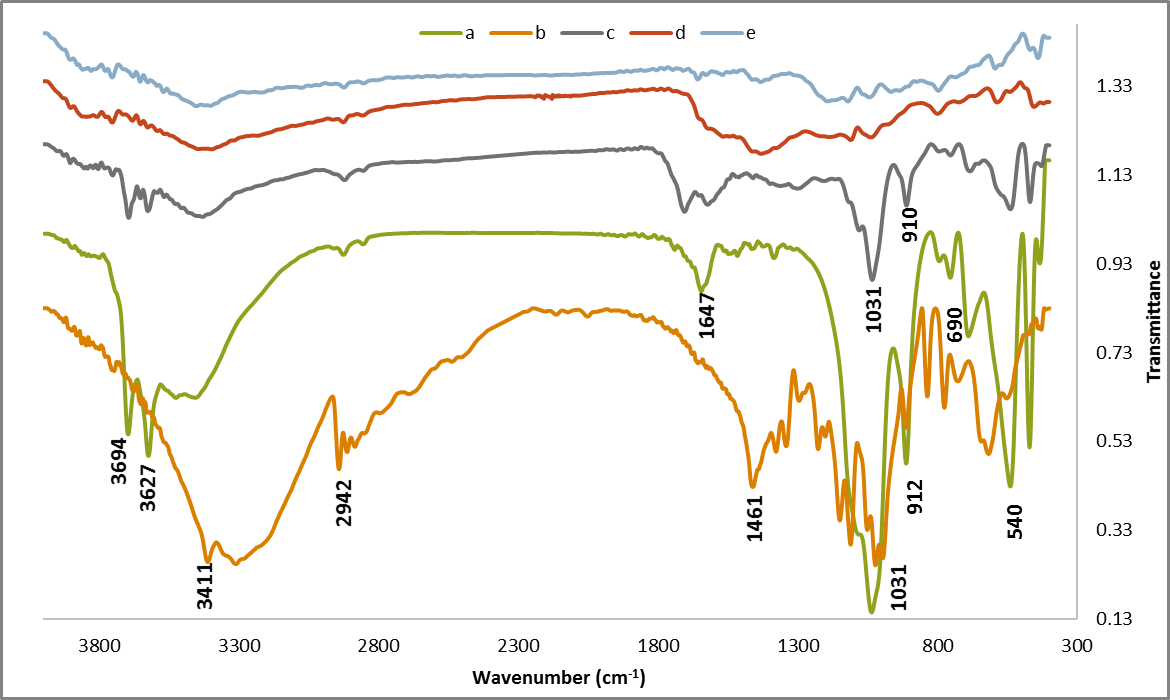


**Figure S2.** The FTIR spectra of (a) pristine Hal, (b) pure glucose, (c) Hal@Glu, (d) Pd@Hal@C and (e) Pd@C.


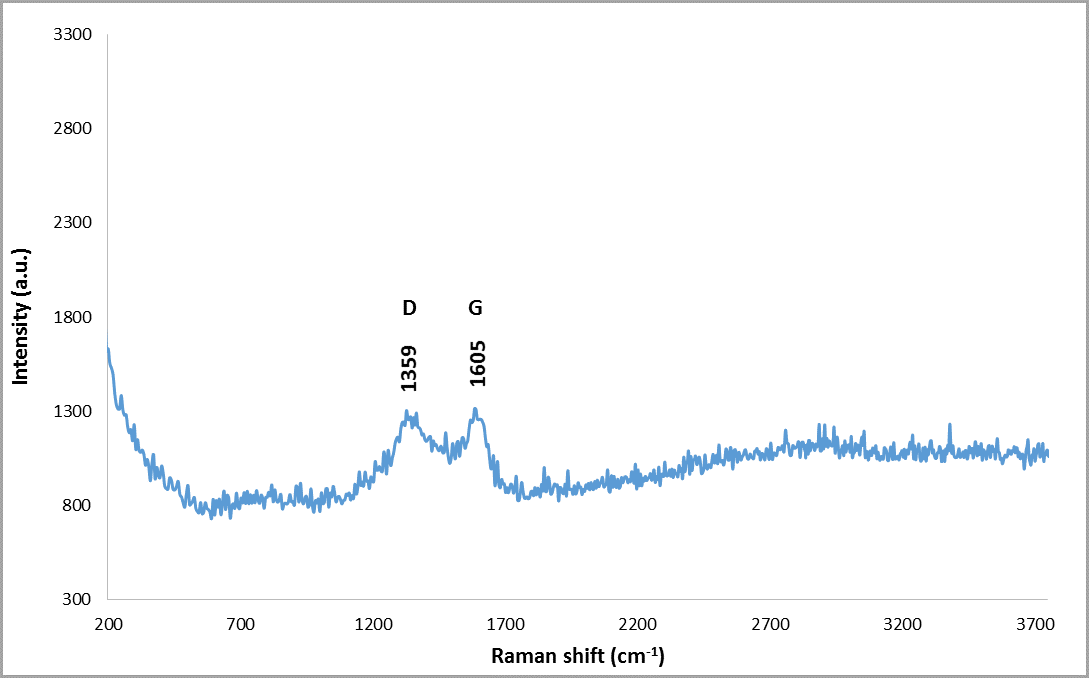


**Figure S3.** The Raman spectrum of the Pd@C.

**
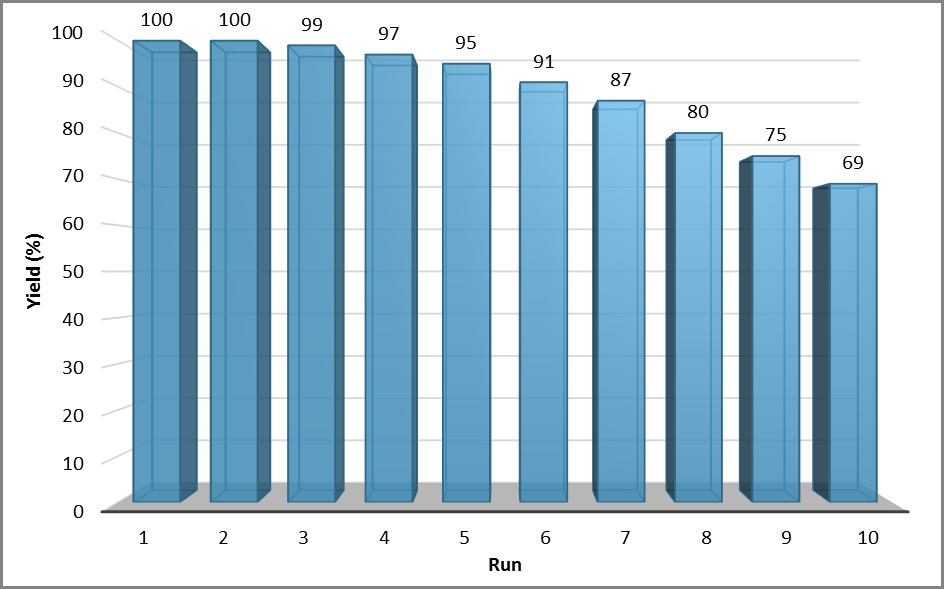
**

**Figure S4.** Recyclability of the Pd@C catalyst in hydrogenation reaction of the model reaction under optimum reaction condition.

| **Table S1**  Textural properties of Pd@C and Pd@Hal@C. | | |
| --- | --- | --- |
| **Sample** | **S_BET_ (m^2^/g)^a^** | **V_t_ (m^3^/g)^b^** |
| Pd@C | 1761 | 404.6 |
| Pd@Hal@C | 132 | 3.0 |
| ^a^ S_BET_ (BET surface areas).  ^b^ V_t_ (total pore volume was estimated at a relative pressure of *P/P_0_*=0.990. | | |
